# The effect of group art therapy on the stress coping ability of Chinese international students in South Korea: using the person-in-the-rain test

**DOI:** 10.3389/fpsyg.2024.1387847

**Published:** 2024-06-27

**Authors:** Yue Yin, Kyung Soon Ko

**Affiliations:** ^1^Kunming University, Kunming, China; ^2^Department of Creative Arts Therapy, Jeonju University, Jeonju, Republic of Korea

**Keywords:** group art therapy, stress coping, Chinese students, PITR, study abroad

## Abstract

**Introduction:**

This study aimed to determine the effectiveness of group therapy on the stress coping skills of Chinese students in Korea using a pre-test post-test control group design.

**Methods:**

Thirty participants were randomly placed into an experimental group (EG; *n* = 15) and a control group (CG; *n* = 15). The EG completed eight 120-min sessions of group art therapy. The Person-in-the-Rain (PITR) drawing test was conducted with both groups before and after the EG completed art therapy.

**Results:**

The test revealed that EG members demonstrated lower stress and significantly higher stress resources and coping abilities than the CG members after the intervention. The group art therapy program improved the EG participants’ ability to deal with stress.

**Discussion:**

This study’s findings may offer insights useful for determining how best to provide psychological and emotional support to international students who have left their home countries and are continuing their academic journeys abroad.

## 1 Introduction

Globalization has encouraged and enabled more students to study abroad (Hwang, 2017; Luo, 2022; Yang, 2022). In 2004, the Korean government launched the “Study Korea Project” to attract international students; as a result, the number of foreign students enrolled in courses at domestic educational institutions increased to approximately 160,000 by 2019 (Hwang, 2019). According to the Korean Education Statistics Service (2022), there were 166,892 foreign students residing in Korea, including 67,439 (44.2%) from China.

An “international student” is a person who resides in another country to complete formal studies at an educational institution (Dunne, 2009; Yoo, 2011). Along these lines, Sovic (2008) states that international students’ transitions from one country to another may involve positive and negative emotions. Notably, the process of adapting to a new country can have social, cultural, and academic effects on students that may significantly impact their achievements. Additionally, international students must leave their families and other members of their social support networks behind (Werkman, 1980; Walton, 1990). Sovic (2008) states that these kinds of transitions are closely associated with the concepts central to Bowlby’s (1973) attachment theory. Given that the number of Chinese students in Korea has tremendously increased in recent years, various studies have been conducted on the causes of their stress.

Some Chinese students in Korea, who have left their home country and cultures, experience some difficulties in adapting to their new culture and problems with studying (Hu, 2011; Hoang, 2021) due to cultural discrimination, homesickness (Hoang, 2021), financial burdens, interpersonal difficulties, family expectations, identity development tasks (Cho and Jon, 2009; Xu, 2021), environmental changes, role changes, identity confusion, perceived discrimination, cultural conflicts, and inconveniences (Park et al., 2010).

Burns (1991) states that “stress occurs when the individual believes that they cannot meet the demand being made on them by the environment” (p. 67). The stresses of cultural adjustment are accompanied by physical, psychological, social, and identity confusion as well as anxiety, depression, isolation, and somatization (Ju and Kim, 2017). The stress that occurs during the cultural adjustment process can negatively affect depression and daily life in general. For example, 50% of Chinese international students who visited a school counseling center were depressed, mainly due to cultural adjustment and academic stress (Zhao, 2016). Along these lines, cultural adjustment stress among Chinese students in Korea has been linked to academic stress (Kim, 2007; Kim, 2010). Multiple stresses have been associated with mental health problems, such as anxiety and depression (Koung and Jang, 2010). There seems to be a benefit in providing emotional and psychological support to those who need it during their academic journey abroad.

Art therapy facilitates free exploration and creativity, allowing participants to express things that they may find difficult or impossible to express verbally by enabling them to respond to defense mechanisms for ambiguity and confusion with relative ease (Rubin, 2011; Greenberg, 2017). Creating art can be an effective way to encourage people who experience psycho-emotional difficulties, including those who may find it difficult to directly express their emotions, thoughts, or feelings (Wadeson, 1987; Malchiodi, 2005).

Interactions between participants in group art therapy can make art therapy a way to examine lifestyles and explore interpersonal relationships (Park, 2020). Yalom (1995) argues that the forces and energies generated in a group interact in complicated ways and suggests that group cohesion compels actions, thoughts, emotions, and experiences that can energize group members. Through this process, group members gradually gain a deeper understanding of themselves, and these changes can persist even after the group no longer meets.

As noted above, the rising number of Chinese international students in Korea has inspired related studies. In particular, existing studies suggest that art therapy can be a means of self-expression for international students who may have trouble expressing themselves due to the influence of cultural collectivism. For example, previous studies have found that expressive enhancement (Cho, 2022), sense of belonging, emotional support (Li and Han, 2019), and the expression of repressed emotions (Park and Lee, 2016) can reduce stress.

These findings suggest that social and emotional support programs are necessary to help international students cope with stress. However, no studies have yet been conducted on whether group art therapy affects international students’ abilities to cope with stress. As completing an academic degree abroad usually takes at least a couple of years, it is important to explore whether there is the possibility of using art therapy as a verbal and non-verbal method to help Chinese international students cope with stress during school.

This study sought to uncover the benefit of group art therapy on the abilities of Chinese students studying in Korea to cope with stress. Specifically, we provided group art therapy to an experimental group (EG) and studied the group members’ abilities to cope with stress after the therapy against the stress coping ability of a control group (CG). We measured the ability to cope with stress using the Person-in-the-Rain (PITR) drawing test. Through this study, we hope to find a way to support Chinese students who have left their home countries for academic purposes by determining how to best help them cope with stress in their new environment and culture. The following hypothesis was tested: group art therapy will significantly increase the stress coping ability of Chinese students in Korea.

## 2 Materials and methods

### 2.1 Study design

In this study, a pre-test post-test control group design was used to investigate the impact of eight sessions of group art therapy on the stress coping ability of international students studying in Korea (Table 1). We used the PITR drawing test to measure the students’ coping abilities.

**Table 1:** Study design.

Participants	Pre-test	Group art therapy	Post-test
EG (*n* = 15)	Q_1_	X	Q_2_
CG (*n* = 15)	Q_3_	–	Q_4_

Q_1,_ Q_3_, pre-test (PITR); X, eight sessions of group art therapy; Q_2_ Q_4_, post-test (PITR); −, no intervention.

### 2.2 Participant recruitment

To recruit participants for the experiment, the purpose of the study and the criteria for participation were posted on an online chat room (WeChat). Among the applicants, 30 participants who met the recruitment criteria were selected. The inclusion criteria were as follows: (1) Chinese student studying in Korea and currently residing in Korea, (2) currently completing a university or graduate degree; (3) experiencing stress. Regarding the participants’ proficiency in Korean as assessed by the Test of Proficiency in Korean (TOPIK), in the experimental group (EG), 12 students (80.0%) scored at level 2 or below, while 3 students (20.0%) scored at level 3 or above. Moreover, 10 students (66.7%) below level 2 had no recorded TOPIK grade. In the control group (CG), 10 students (66.7%) scored at level 2 or below, and 5 students (33.3%) scored at level 3 or above. Additionally, 9 students (60.0%) below level 2 had no recorded TOPIK grade. It should be noted that all participants were required to achieve at least a level 4 proficiency to graduate from university. The demographic information of the participants is shown in Table 2.

**Table 2:** Demographic characteristics of participants.

Domain	Area	EG (*n* = 15)		CG (*n* = 15)		Total	
		*n*	%	*n*	%	*N*	%
Sex	Male	10	66.7	12	80.0	22	73.3
	Female	5	33.3	3	20.0	8	26.7
Residency (months)	<12	4	26.7	6	40.0	10	33.3
	≥12	11	73.3	9	60.0	20	66.7
TOPIK level	Under 2	12	80.0	10	66.7	22	73.3
	Over 3	3	20.0	5	33.3	8	26.7
Major	Arts	10	66.7	2	13.3	12	40.0
	Natural sciences	5	33.3	5	33.4	10	33.3
	Humanities and social sciences	0	0.0	8	53.3	8	26.7
Degree	BA and MA	7	46.7	4	26.7	11	36.7
	Doctorate	8	53.3	11	73.3	19	63.3

### 2.3 Measurement: person-in-the-rain drawing test

The PITR test was devised by Arnold and Abraham (Hammer, 1958) and is used to determine whether the test taker can cope with stress. Specifically, the test taker uses pencil drawings to convey information about their level of stress and their ability to cope with it. The amount, direction, and intensity of rain depicted in the picture show the participants’ subjective perception of their amount of stress, and the objects that protect the figure, such as umbrellas, raincoats, and boots; the facial expressions; the position of the figure; and the size of the figure are interpreted as stress coping resources (Jue, 2019). In this study, we used the Korean version (Lee, 2008) of Lack’s (1996) PITR-SRC interpretation method to score the participants’ drawings.

The PITR questions consist of 35 items within three domains (see Table 3). The stress domain (S) focuses on stress levels and includes 16 items, S1–S16. The resource domain (R) focuses on stress resources and includes 19 items, R1–R19. The coping domain (C) focuses on the ability to cope with stress and includes 3 items. If the coping ability score is positive (+), then there are more resources than stress and thus relatively little stress. Meanwhile, if the coping ability score is positive (+), then a negative number (−) indicates more stress than resources.

**Table 3:** PITR scoring scale (Lack, 1996).

**Domain**	**Item**	**Scoring standard**
Stress (S) = Sum of S1–S16	S1–S8	Existence: 1 point, none: 0 points (per question)
	S9–S16	# of figures (1 point)
Resource (R) = Sum of (R1–R16)–(R17–R19)	R1–R16	Existence: 1 point, none: 0 point (per question)
Coping (C) = Sum of R–S	R17–R19	Existence: 1 point, none: 0 point (per question) except, R18, which is scored by # of figures (1 point)
	Score of R – Score of S	

### 2.4 Procedure of facilitating the PITR test

The participants completed the PITR drawing test before and after the EG participated in a group art therapy program. In order to conduct the PITR test, the researcher provided the participants with A4 paper (210 × 297 mm), a 4B pencil, and an eraser and asked them to draw a person in the rain for 15 min. Next, the researcher advised the participants as follows: “It’s raining. Draw a person in the rain. At this time, please draw the person in full form, not in the shape of a cartoon or a stick.” The researcher allowed the participants to draw freely; that is, the researcher did not advise participants on things such as the size and shape of the drawing or the position of the figure. When participants asked the research questions about how they should draw while completing the test, the researcher responded, “You can draw whatever you want.” Finally, we collected qualitative data in written form by asking participants who they drew, what the person in their drawing was doing, and how the person in their drawing felt. This was scored as supplementary data for the PITR picture analysis index. In particular, if it is not a picture of only one person, when scoring R11-R15, it was scored by referring to the answer of “Who is the main character in the picture?” (R11-Whole face; R12-Smile; R13-Centred figure; R14-Size of figure; R15-Full figure).

### 2.5 Procedure of grading of the PITR test

To ensure the reliability of the PITR scoring procedure and results, three graders, including researchers, scored the tests. Before scoring, 60 original copies and copies of the pre- and post-test PITR results from the EG and CG, scoring guidelines, and scoring tables were given to the graders. To minimize grader bias, 60 copies of the PITR test results were randomly numbered so that participants could not be identified. A Chinese interpreter was included to facilitate communication between graders while they scored the tests.

Before scoring, the researcher conducted a test to ensure the graders understood the analysis criteria for each item in the test. After a thorough discussion of the scoring system and criteria, each rater independently scored all 60 tests to ensure reliability. In addition, to ensure consistency, the graders evaluated the items when the grading results were checked against each other. A Pearson correlation analysis was performed to analyze the raters’ comparative confidence levels (see Table 4).

**Table 4:** Validity check between graders for the PITR.

**Grader**	**Stress**	**Resource**	**Coping**
A and B	0.782^**^	0.843^***^	0.810^***^
B and C	0.936^***^	0.982^***^	0.952^***^
A and C	0.814^**^	0.823^***^	0.811^***^

*p < 0.05, **p < 0.01, ***p < 0.001.

**Figure 1:**
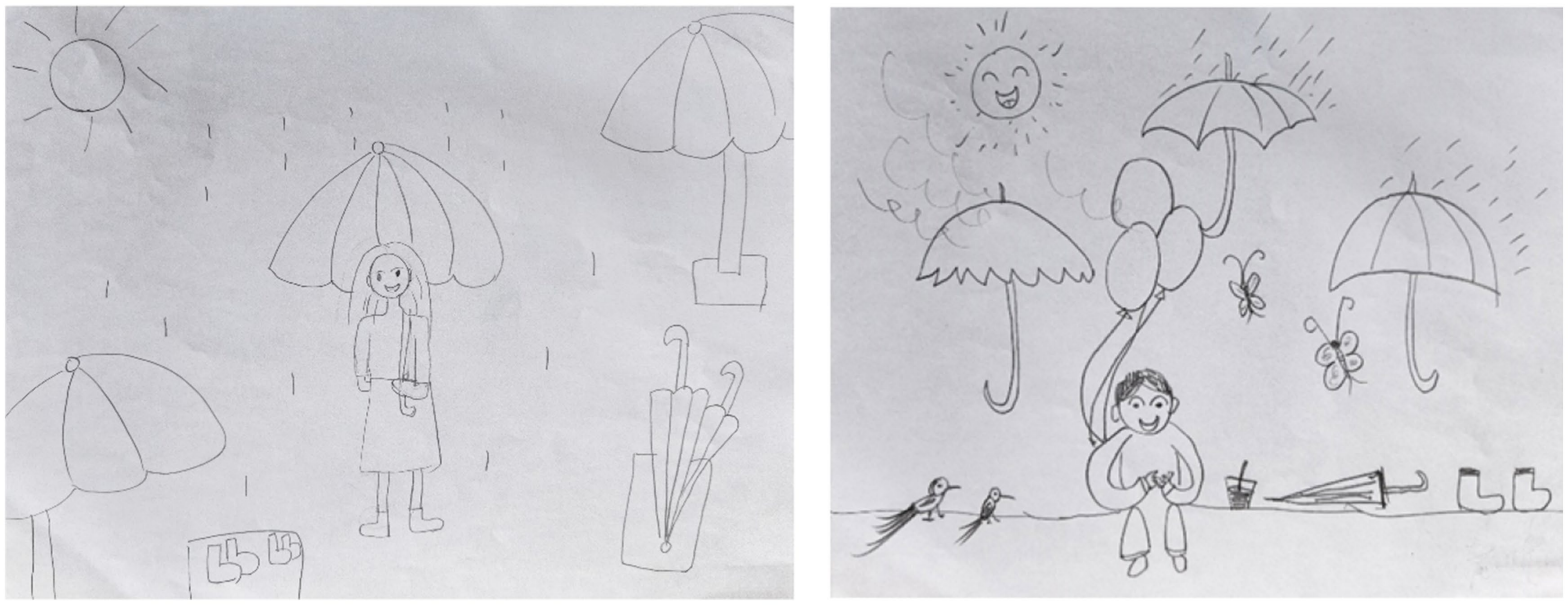
Representative participant’s PITR drawing, scored from 0 to −9.

**Figure 2:**
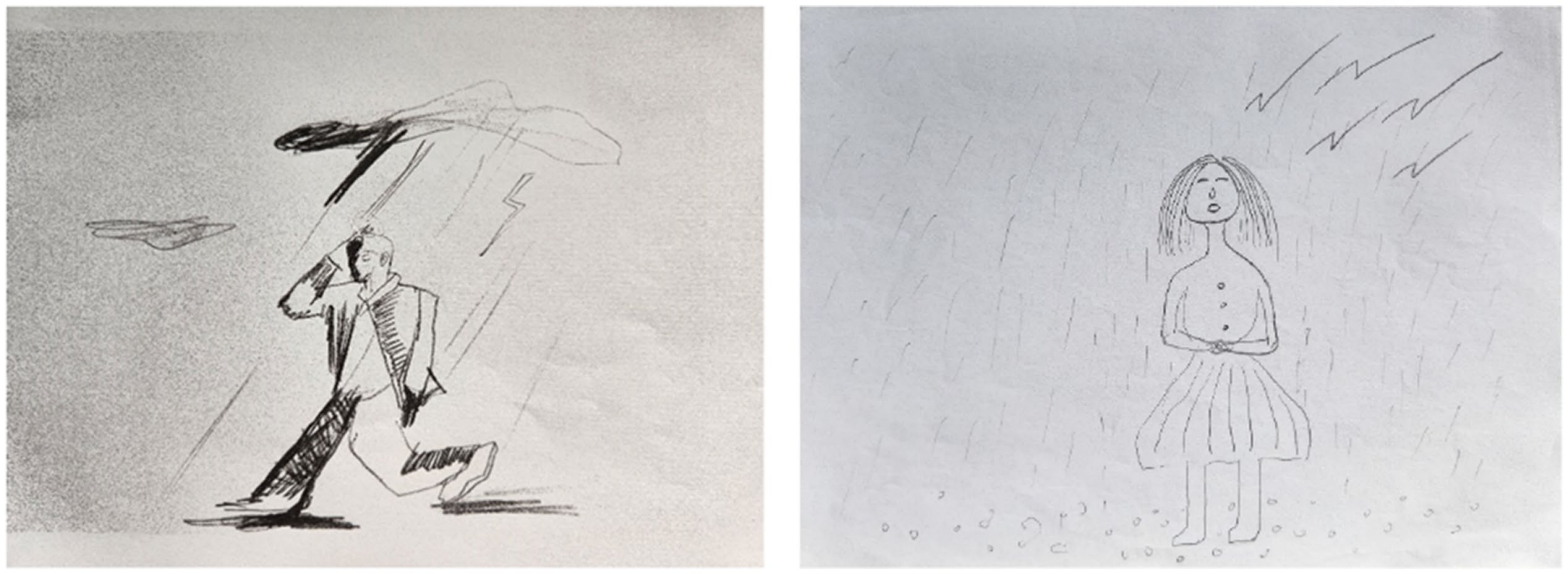
Representative participant’s PITR drawing, scored from −10 to −1.

**Figure 3:**
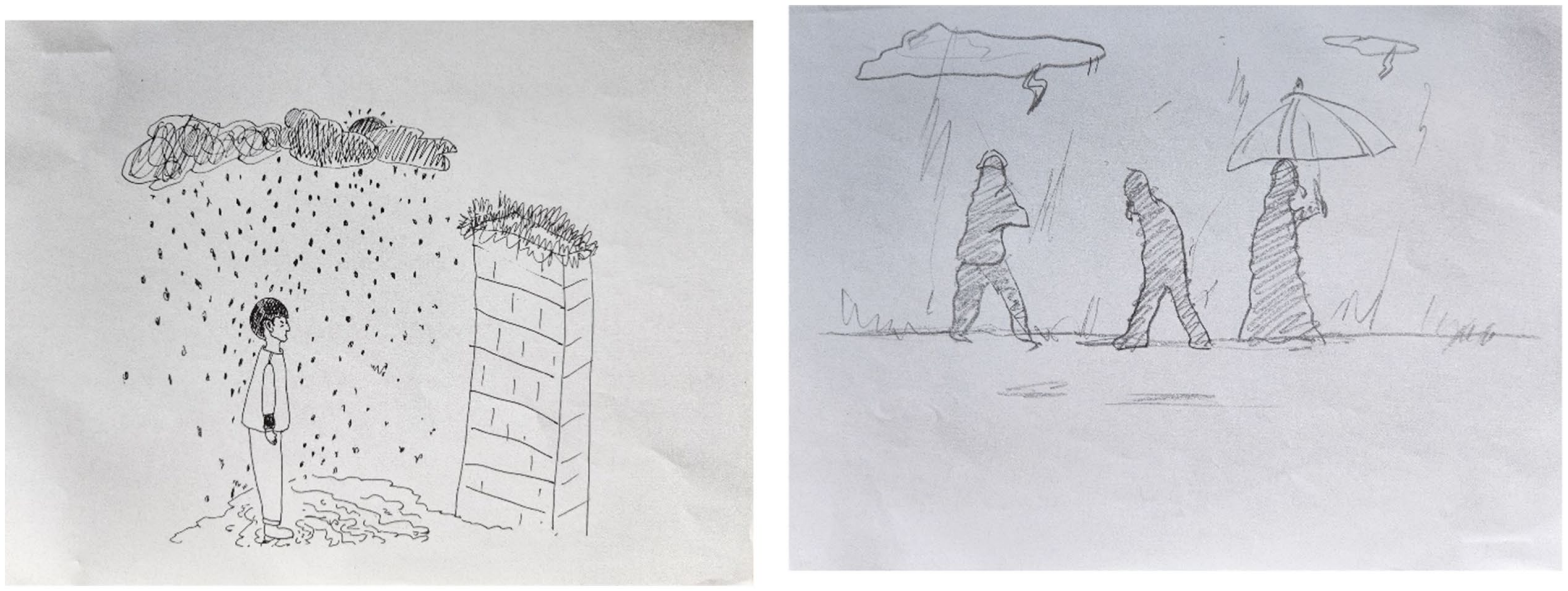
Representative participant’s PITR drawing, scored from −20 to −11.

**Figure 4:**
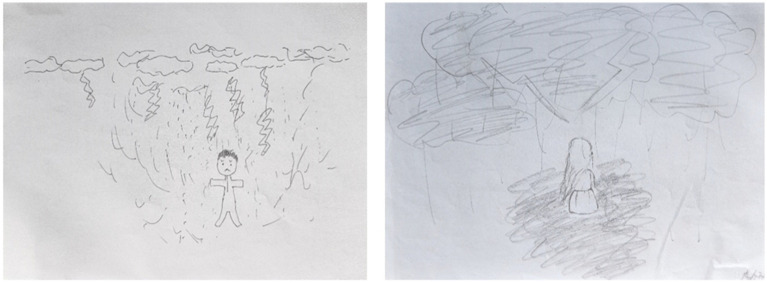
Representative participant’s PITR drawing, scored from −30 to −21.

Raters A and B had a high “stress” confidence correlation coefficient of 0.782, a “stress resources” confidence correlation coefficient of 0.843, and a “coping ability” confidence correlation coefficient of 0.810. Meanwhile, Raters B and C had a “stress” confidence correlation coefficient of 0.936, a “stress resource” confidence correlation coefficient of 0.982, and a “coping ability” confidence correlation coefficient of 0.952. Lastly, Raters A and C had a “stress” confidence correlation coefficient of 0.814, “stress resources” confidence correlation coefficient of 0.823, and a “coping ability” confidence correlation coefficient of 0.811. These results suggest that the three graders were fully familiar with the scoring criteria and independently graded according to these clear criteria (see Figures 1–4).

### 2.6 Intervention

The 30 Chinese international student participants living in Korea were randomly divided into the EG (*n* = 15) and CG (*n* = 15). As established above, the EG completed an eight-session group art therapy program facilitated by an experienced Korean art therapist and the CG did not complete this program. Each session of the therapy program was 120 min long (this length was based on interpretation time and group size) and comprised three phases: the introductory phase (20 min), the task and activity phase (60 min), and the organizing and verbal sharing phase (40 min). The EG’s program consisted of themes related to stress, such as emotional suppression, barriers, cultural adaptation stress, academic stress, self-acceptance, positive images, and future vision after reviewing literature and consulting with a therapist experienced with diverse cultures. Table 5 shows intervention.

**Table 5:** The themes and goals of each art therapy session.

**Session #**	**Theme**	**Goals**
1	Self-introduction	Building rapport and group cohesion
2	My culture	Cultural identity ·supportive experiences from group members
3	Difficulties I am facing	Culture and expression of clash ·sharing difficulties during acculturation
4	Five facial expressions	Awareness of my emotions and expressions
5	Barriers in front of me	Expression of academic stress and empathetic response Problem solving to relieve academic stress
6	Magical lamp and miracle question	Explore inner desires and expressions Supportive from group members
7	My name card	The life I want to build and my self-image Explore inner resources and motivation
8	A gift box for myself	Internalize a positive self-image

### 2.7 Data analysis

The data were analyzed as follows: SPSS 26.0 was used for statistical processing. A frequency analysis was performed to assess the demographic characteristics of the participants. Mann–Whitney U tests were performed to test the homogeneity of the EG and CG before the program was implemented. A comparative analysis was performed based on the PITR-SRC (Lack, 1996) scoring method developed by Lee (2008) and reliability tests were performed to determine if the raters’ scoring results matched. A Wilcoxon signed-rank test was performed to check for differences in scores between the groups. Finally, participants’ nonverbal expressions, verbal expressions, and changes were analyzed in the context of the PITR.

### 2.8 Ethical considerations

This study is part of the author’s doctoral dissertation (Yin, 2023) and its description has been modified to align with the journal format. This study was approved by the Institutional Review Board (IRB) of the Jeonju university (JJIRB-220421-HR-2022-0407). The researcher advised the participants on the purpose of the study, their right to confidentiality, and their right to stop participating in the study without any penalty. All data were gathered after IRB approval and participant consent. Since a Korean art therapist facilitated the program, a Chinese interpreter was present during the sessions to facilitate accurate communication. All data were stored on the researcher’s personal computer with a password to protect privacy.

## 3 Results

### 3.1 Verification of the homogeneity between the experimental and control groups

The Mann–Whitney *U* test was conducted to determine the homogeneity of the EG and CG. As a result of the homogeneity test of the means of the EG and CG, the average score for the “stress” domain in the PITR test among the EG was 14.67 points; while this is higher than the CG’s score of 11.87 points, this difference was not statistically significant (*p* > 0.05). Meanwhile, the mean score for “stress resources” was higher in the EG (2.13) than in the CG (0.33); however, these results were also not statistically significant (*p* > 0.05). Regarding “coping ability,” the EG demonstrated a lower mean score (−12.53) than the CG (−11.53); again, these results were not statistically significant (*p* > 0.05). Therefore, the homogeneity of the PITR test between the two groups was confirmed (see Table 6).

**Table 6:** Verification of homogeneity between the experimental and control groups.

Domain	EG (*n* = 15)		CG (*n* = 15)		Z
	*M*	SD	*M*	SD	
Stress (S)	14.67	3.416	11.87	5.817	−2.206
Resource (R)	2.13	4.502	0.33	3.904	−1.334
Coping (C)	−12.53	5.041	−11.53	7.990	−0.166

### 3.2 Comparison of the experimental group and control group PITR results

In the post-test conducted after the group art therapy, a Mann–Whitney U analysis was performed to identify the differences in the post-test results of the EG and CG. The results are shown in Table 7. Specifically, the comparison indicated statistically significant differences across all areas. In the domain of “stress,” the EG’s mean score (5.27) was lower than the CG’s mean score (15.73) (*p* < 0.001). Regarding “stress resources,” the EG’s mean score (6.93) was higher than the CG’s main score (−0.33) (*p* < 0.001). In terms of “coping ability,” the EG’s mean score (1.67) was statistically significantly higher than the CG’s mean score (−16.07) (*p* < 0.001). Therefore, the pre-post test results reveal that the group art therapy program significantly improved the EG’s stress-related capacities compared to the CG.

**Table 7:** PITR results for the experimental and control groups.

Domain	EG (*n* = 15)		CG (*n* = 15)		Z
	*M*	SD	*M*	SD	
Stress (S)	5.27	2.120	15.73	5.257	−4.603***
Resource (R)	6.93	4.383	−0.33	3.519	−3.627***
Coping (C)	1.67	4.012	−16.07	6.076	−4.633***

***p < 0.001.

### 3.3 Comparison of the experimental group and control group PITR pre and post results

To analyze the effect of the group art therapy program on Chinese international students in Korea, we compared the pre-post and post-test PITR results of the two groups. A Wilcoxon signed-rank test was conducted to determine whether the pre- and post-test changes were statistically significant (see Table 8).

**Table 8:** Differences in the pre- and post-test results of the experimental group.

**Domain**	**Pre**		**Post**		***Z***
	***M***	**SD**	***M***	**SD**	
Stress (S)	14.67	3.416	5.27	2.120	−3.415**
Resources (R)	2.13	4.502	6.93	4.383	−2.702**
Coping (C)	−12.53	5.041	1.67	4.012	−3.411**

**p < 0.01.

The pictures in the EG for the post-test usually appeared to have no rain or a dot shape, and the protagonist in the picture had no contact with the rain. There was not enough wind, puddles, or lightning. Most of the pictures were cloudless or appeared to have friendly clouds. There were also many pictures in which there was appropriate protection, and one or more of the protections were depicted. The impressions of the picture appeared to be positive, with low stress scores and high stress coping resource scores. In the case of the CG, most of the pictures showed a lot of rain, or rain was drawn in various shapes such as dots, lines, circles, and drops. In almost all the paintings, the protagonist was shown to be in direct contact with the rain. Wind, puddles, lightning, and dark clouds were also depicted a lot, and the stress was high. In addition, there was no protection or it appeared inappropriately; the protagonist had also omitted body parts or only the back of the character was shown. The protagonist in the picture looked tired and stressed, and depicted exuded a negative vibe. The stress coping resources were low (see Table 9).

**Table 9:** Differences in the pre- and post-test results of the control group.

Domains	Pre		Post		***Z***
	***M***	**SD**	***M***	**SD**	
Stress (S)	11.87	5.817	15.73	5.257	−2.332*
Resource (R)	0.33	2.904	−0.33	3.519	−0.126
Coping (C)	−11.53	7.990	−16.07	6.077	−1.652

*p < 0.05.

**Figure 5:**
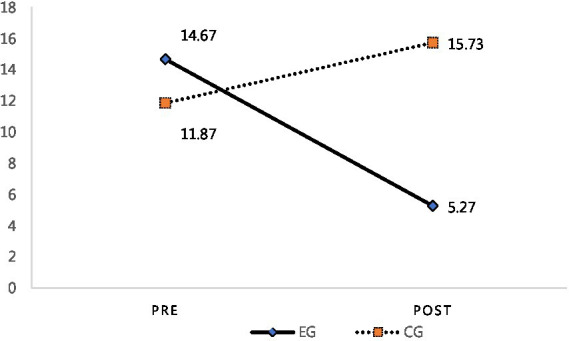
Pre- and post-test stress scores for the EG and CG.

The comparison revealed that the score for “stress” significantly decreased in the EG, as shown in Figure 5. Specifically, the EG’s pre-test mean score of 14.67 decreased to 5.27 in the post-test; this difference is statistically significantly different (*p* < 0.01). Meanwhile, the CG’s pre-test mean score of 11.87 increased to 15.73 in the post-test; this difference is also statistically significantly different (*p* < 0.05) (see Figure 5).

**Figure 6:**
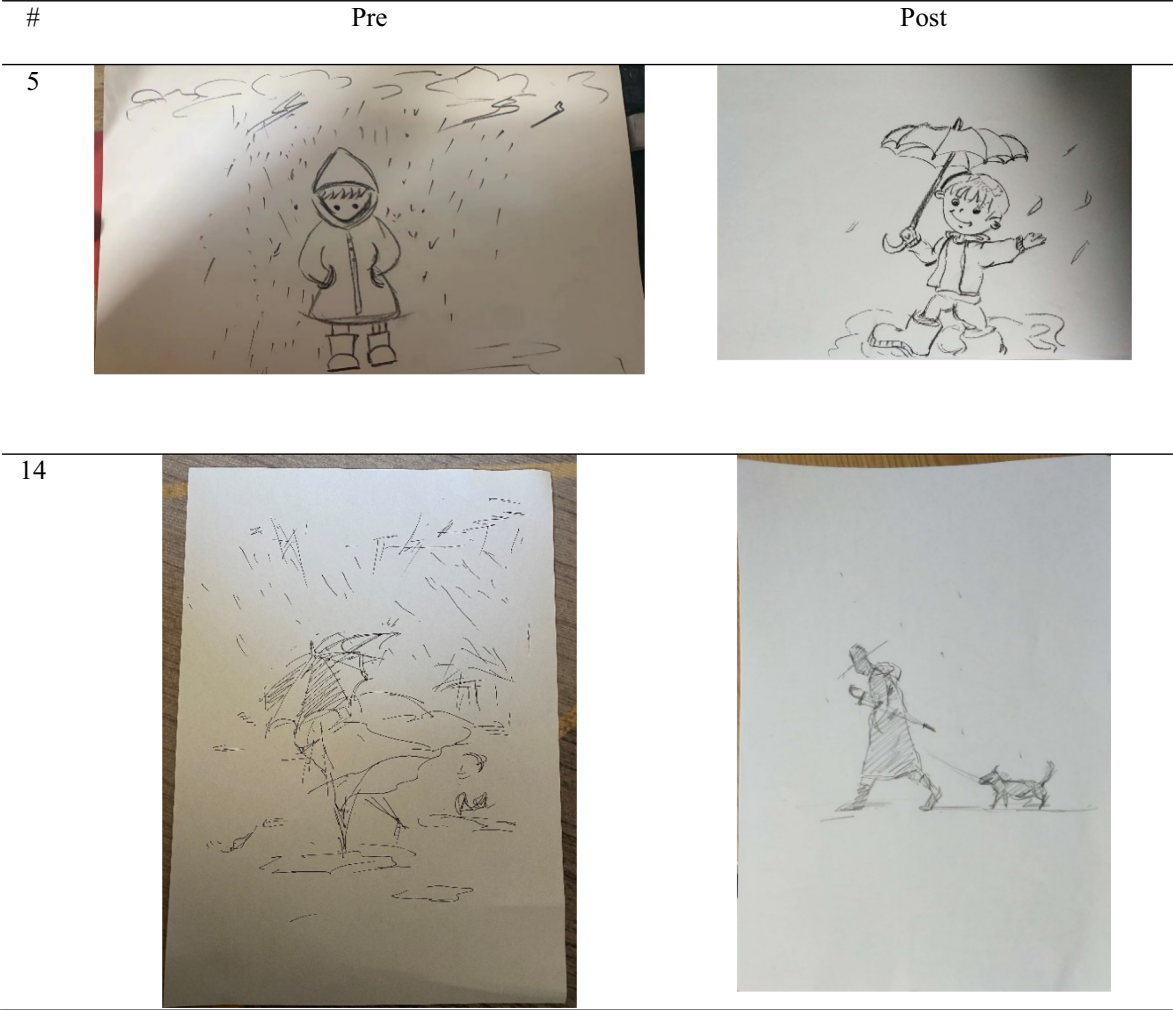
Representative sample PITR drawing from EG, participants 5 and 14.

**Figure 7:**
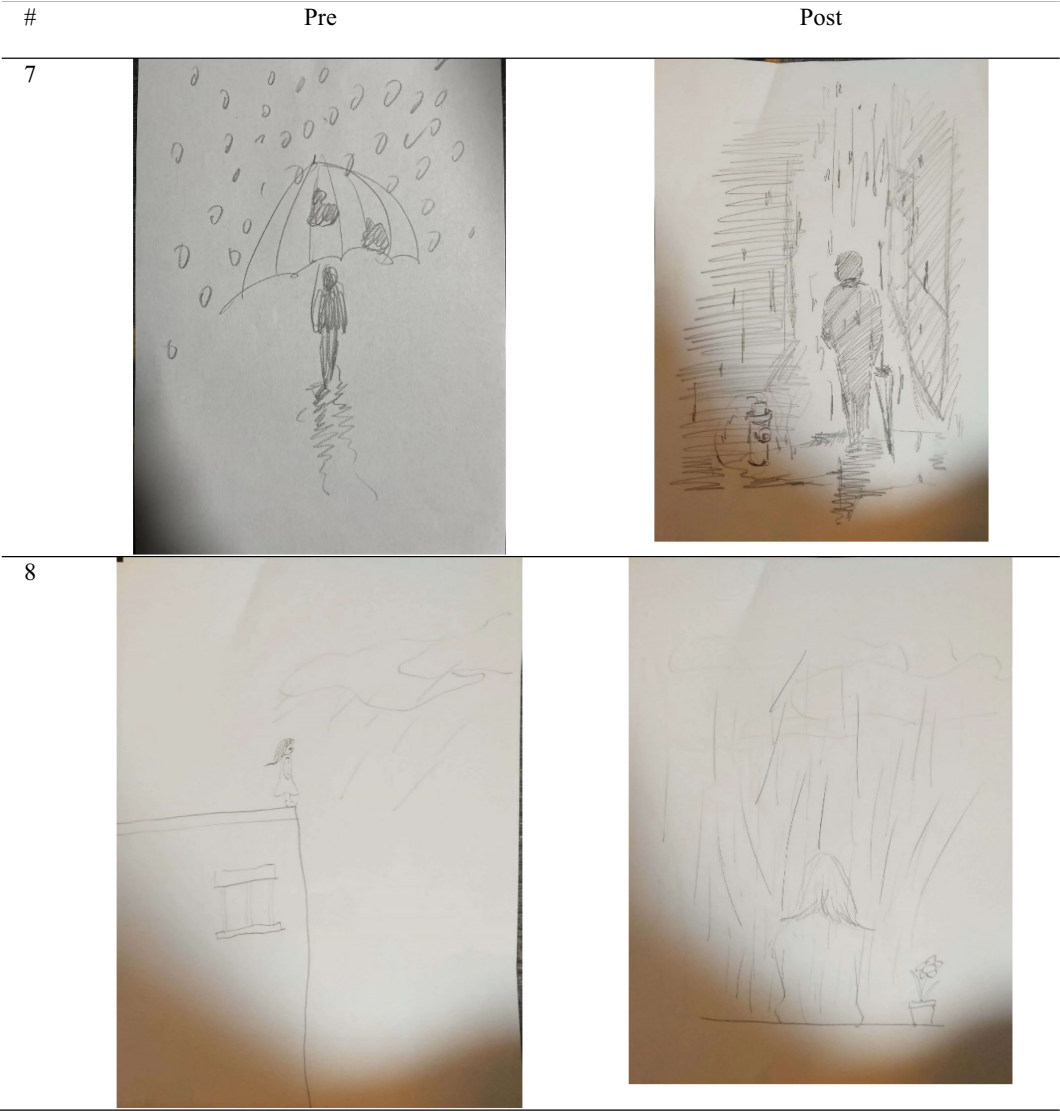
Representative sample PITR drawing from CG, participants 7 and 8.

The stress coping ability of the participants who participated in the group art therapy session was improved by comparing the pre-post of the PITR picture in the actual box group. It seems like the stress was reduced after assessing the items, which comprised a stress measure. Looking closely, the “amount of rain” significantly decreased for all 15 participants from 1 to 15, and the “contact of rain” in the pictures of participants 1, 3, 4, 6, 12, 13, and 14 showed that the “wind” item in the pictures of participants 12 and 14, “hole” item in the pictures of participants 2, 3, 4, 6, 7, 8, 9 and “lighting” item in the pictures of participants 1, 5, 7, 8, 9 disappeared. The “cloud” item in the pictures of participants 3, 4, 5, 6, 8, and 9 showed that the cloud disappeared or the dark cloud became a cloud. It seems that the ability to cope with stress increased throughout the EG through the items, which are measures of coping ability. Looking closely, the pictures of participants 1, 2, 3, 6, 7, 11, and 12 included “protective objects,” and the pictures of participants 3, 12, and 14 showed “protective objects” appropriately so that people in the drawing do not get rained on. The “character” in the pictures of participants 2, 3, 4, 5, 6, 11, and 14 were drawn from the back or side; however, the entire figure could be seen more completely as it was from the front side of human body. Moreover, the main character of the paintings of participants 1, 2, 3, 5, 6, 8, 10, 11, 12, and 15 had a smiling expression. As a result, the main character in the picture turned negative emotions into positive ones (see Figures 6, 7).

**Figure 8:**
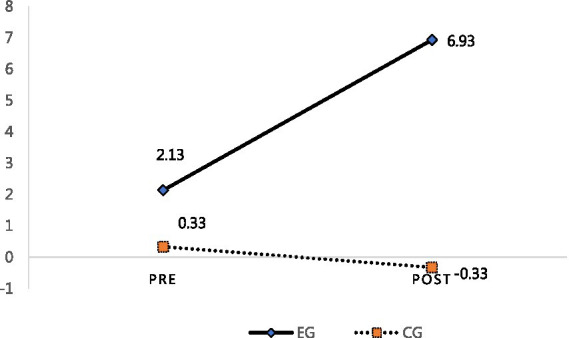
Pre and post-test stress resource scores for the EG and CG.

The “stress resources” of the CG did not change—the group’s pre-test and post-test mean scores for this domain were both 0.33 to a post-test mean score of −0.33; this result accordingly did not reveal a statistically significant difference (*p* > 0.05) (see Figure 8).

**Figure 9:**
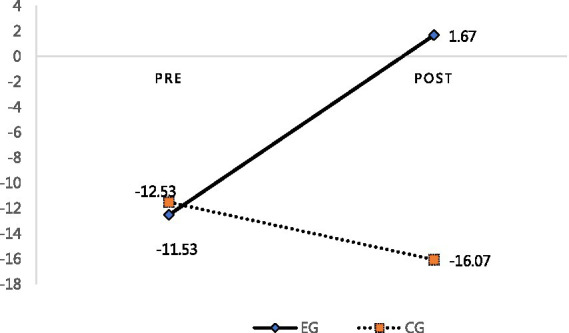
Pre and post-test coping ability scores for the EG and CG.

By comparing the pre-post of the PITR picture in the control group, it was found that the stress and coping ability scales did not change significantly. The stress scale items, namely the “amount of rain,” “contact of rain,” “wind,” “punch,” “lightning,” and “cloud” did not decrease or disappear. The coping ability scale showed that no protection appeared or it was used inappropriately. Furthermore, most of the protagonists in the picture were not complete or appeared in the back. Overall, this showed that the pre-post of the PITR picture in the CG did not change much. Finally, “coping ability” increased in the EG—the EG’s pre-test mean score for this domain was −12.53 and its post-test mean score was 1.67, as shown in Figure 7; this difference was statistically significant (*p* < 0.01). In the CG, the coping ability mean score increased from −11.53 in the pre-test to–16.07 in the post test (*p* > 0.05), which was not significant. Ultimately, these results show that the group art therapy program decreased stress and increased stress resources and coping abilities among Chinese students in Korea (see Figure 9).

## 4 Discussion

The purpose of this study was to determine whether group art therapy could effectively improve the ability to cope with stress among Chinese international students in Korea using a pre-test post-test control group design. The study involved 30 participants who were randomly divided into an EG (*n* = 15) and a CG (*n* = 15). The EG was provided with eight 120 min of group art therapy. The PITR test was conducted with both groups before and after the group art therapy program to measure their scores across different domains of stress; namely: “stress,” “stress resources,” and “coping ability.”

A Mann–Whitney *U* analysis was conducted to compare the results of the PITR between the groups. The EG had lower mean scores for “stress” and higher mean scores for “stress resources” and “coping ability” after the therapy than the CG, and these differences were statistically significant. These results suggest that the group art therapy program improved the international students’ stress coping ability.

A Wilcoxon signed-rank test was conducted to determine the changes in the pre-post test scores for the PITR. The EG’s post-test mean score for “stress” was lower than its pre-test mean score, and its post-test mean scores for “stress resources” and “coping ability” were higher than its pre-test mean scores. Meanwhile, the CG’s post-test mean score for “stress” was significantly higher than its pre-test mean score, its post-test mean score for “stress resources” was the same as its pre-test mean score, and its post-test mean score for “coping ability” was higher than its pre-test mean scores (however, these differences were not significant). These findings may be because the students may have been completing their final exams or dissertations, which would have increased their stress levels.

The findings of this study are consistent with previous research indicating that nonverbal expressive art therapy can reduce stress and enhance emotional stability (Park and Lee, 2016; Cho, 2022). Researchers sought to determine how PITR, as a projective measurement, could benefit international students influenced by East Asian culture. In Western contexts, theories of psychological well-being traditionally emphasize self-actualization, which views human potential through an individual lens (Maslow, 1970). In contrast, Eastern philosophies, especially Confucianism, which profoundly influences Eastern psychology, emphasize cooperation and harmony with others (Kim, 2007). The Western psychoanalytic framework categorizes the self into three layers: the id, the ego, and the superego (Freud, 1923), differing significantly from Asian perspectives. In Asian societies, the concept of self is deeply intertwined with the broader socio-cultural context, including community, family, and social connections, where the self is perceived in relation to society, often merging individual and collective identities (Tseng, 2004; Kim, 2007). However, these concepts are not universally applicable. In East Asian cultures, openly sharing emotional struggles and expressing individuality may seem unfamiliar and challenging (Kim et al., 2008). Thus, PITR could serve as a valuable tool for international students, particularly those from East Asian backgrounds, to become aware of and manage their stress levels effectively within a group setting.

This study had some limitations. First, due to the COVID-19 pandemic limiting access to in-person programs, it was challenging to recruit Chinese students from various universities and a Chinese art therapist in Korea. Consequently, the group art therapy sessions were led by a Korean art therapist over eight sessions, with a Chinese translator present. This resulted in not having a therapist with a Chinese cultural background and language proficiency, as well as a limited number of sessions that may have influenced the study’s results. Second, since participants were recruited on a voluntary basis, we did not screen participants. Third, no follow-up tests were conducted after the participants completed the eight art therapy sessions, thus the persistence of the effects could not be confirmed.

Despite these limitations, this study not only provided mean score differences between the control group and experimental group, indicating the effectiveness of group art therapy, but also incorporated visual data obtained through PITR. These drawings intuitively captured the participants’ stress levels and coping abilities, offering valuable insights as they reflected upon them. The contents of projective examinations such as PITR convey more vivid content than language, which may represent the situation of some international students who cannot express themselves verbally.

## 5 Conclusion

This is particularly relevant for Chinese students studying abroad, as they may be accustomed to not verbally expressing their emotional difficulties due to cultural factors that emphasize community and harmony rather than individual needs. Ultimately, the findings of this study may be applied to enhance the psychological and emotional well-being of Chinese students studying abroad, thereby strengthening their resilience in overcoming stressors during their academic journey.

## Data availability statement

The raw data supporting the conclusions of this article will be made available by the authors, without undue reservation.

## Ethics statement

This study was approved by the Institutional Review Board (IRB) of Jeonju university (JJIRB-220421-HR-2022-0407). The studies were conducted in accordance with the local legislation and institutional requirements. The participants provided their written informed consent to participate in this study. Written informed consent was obtained from the individual(s) for the publication of any potentially identifiable images or data included in this article.

## Author contributions

YY: Writing – original draft. KK: Writing – review & editing.
